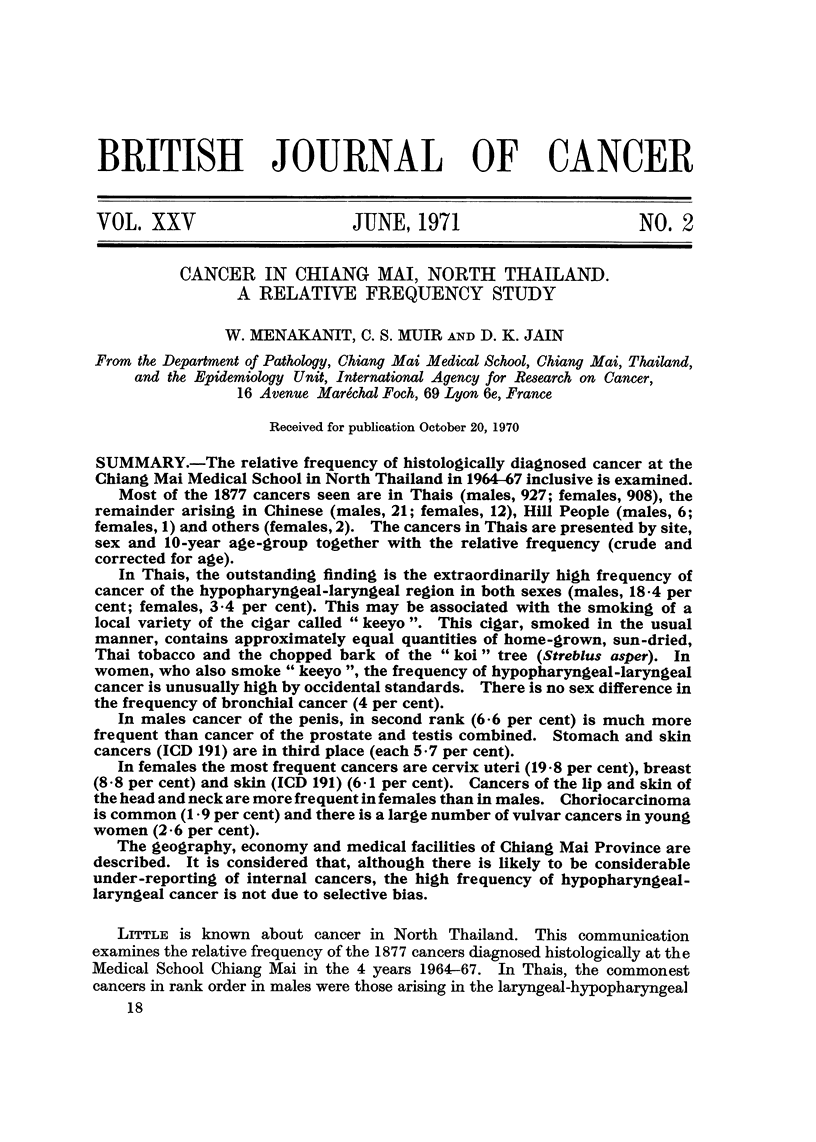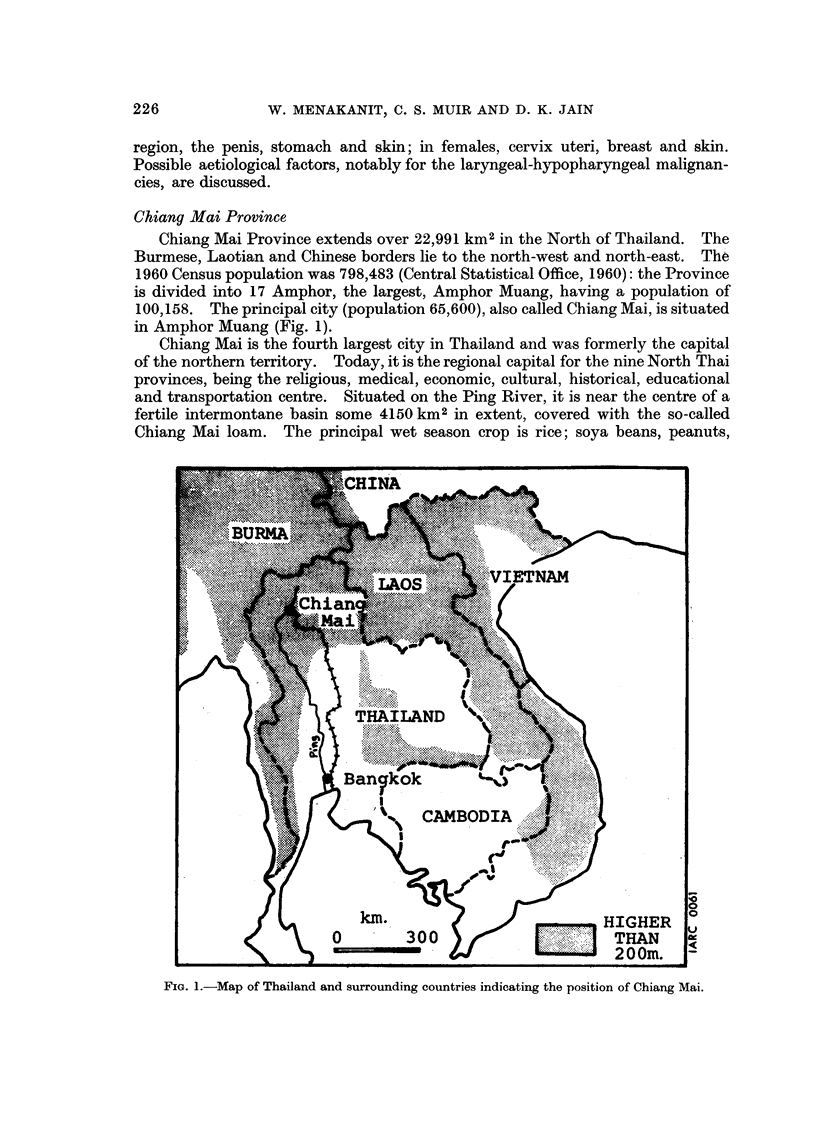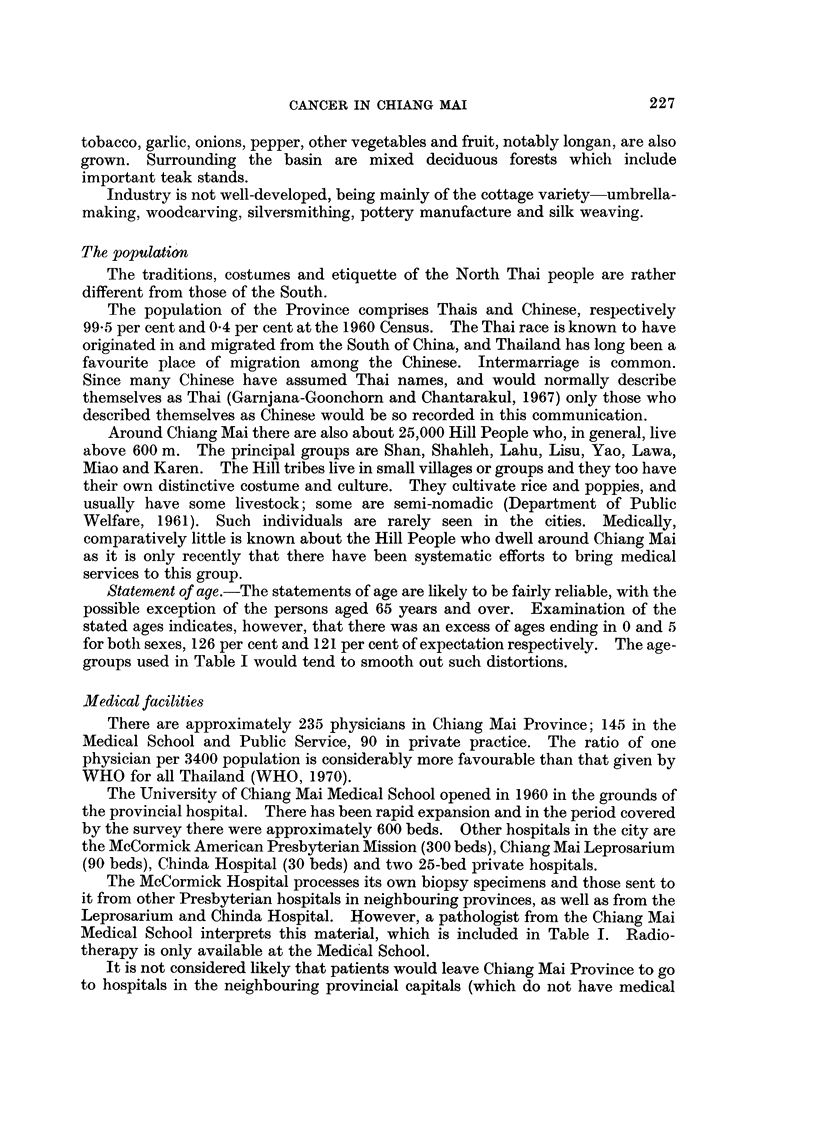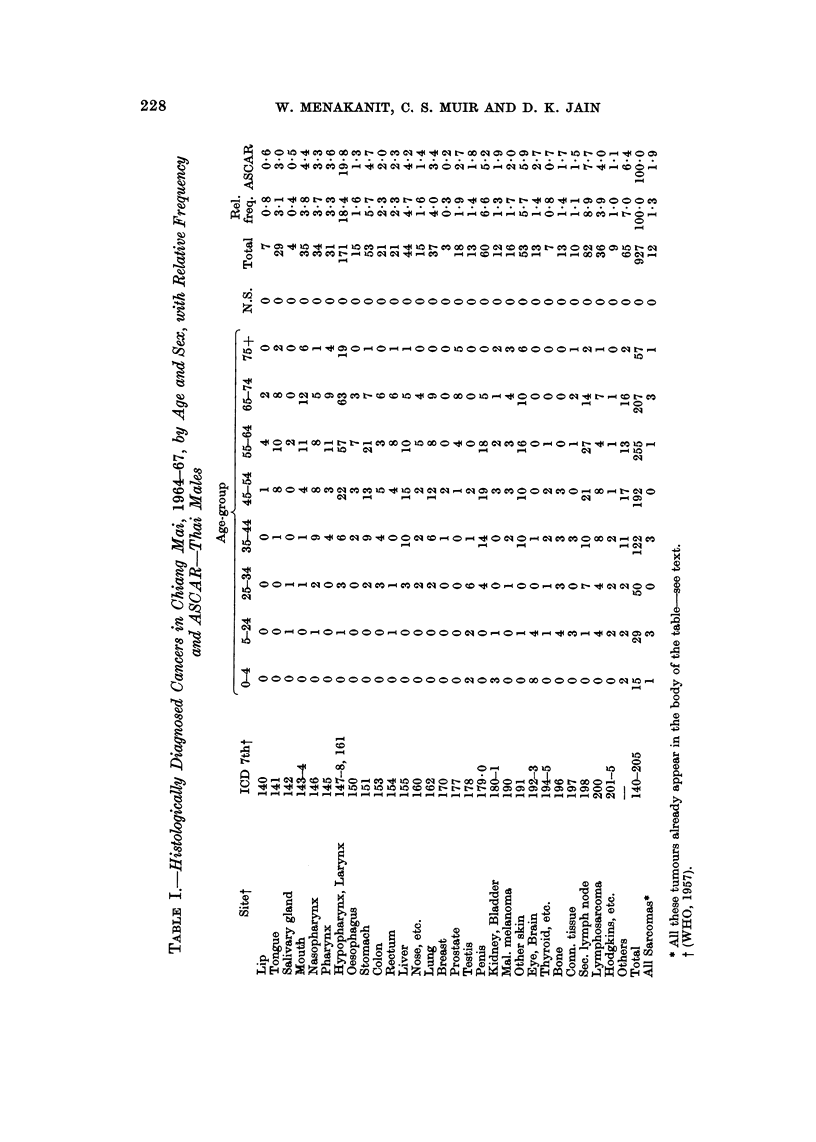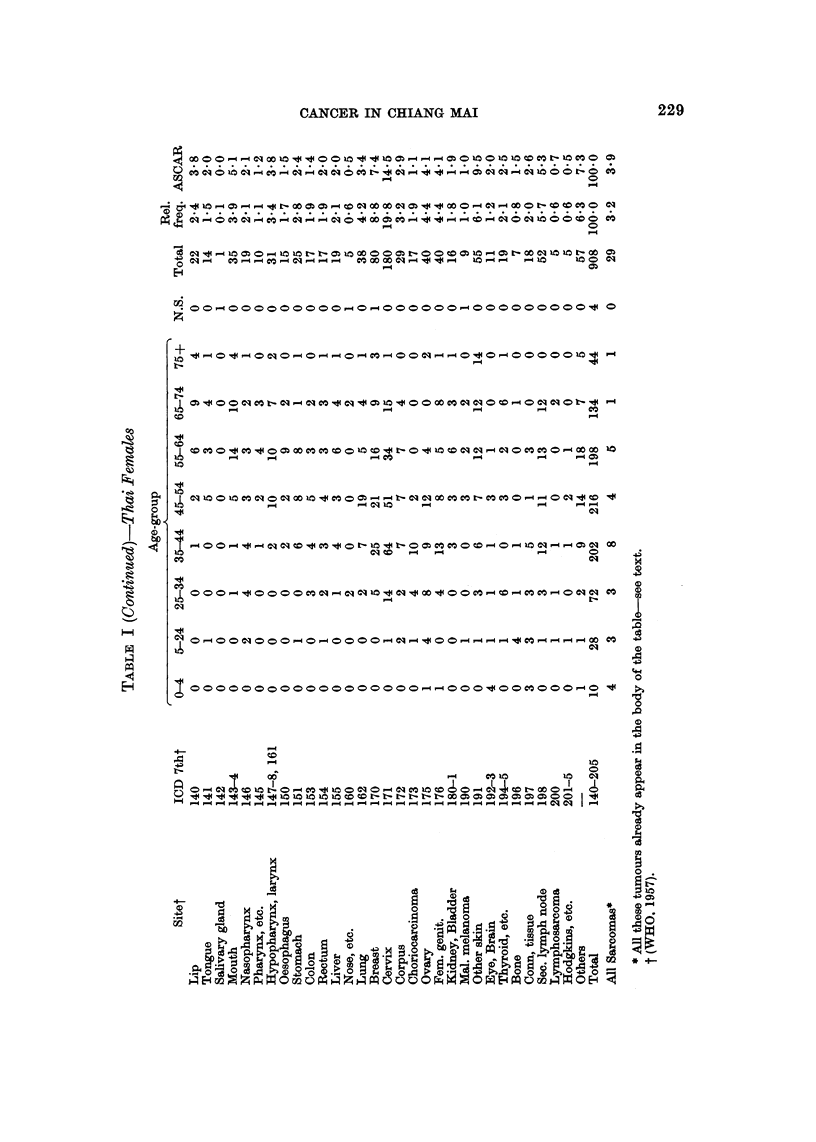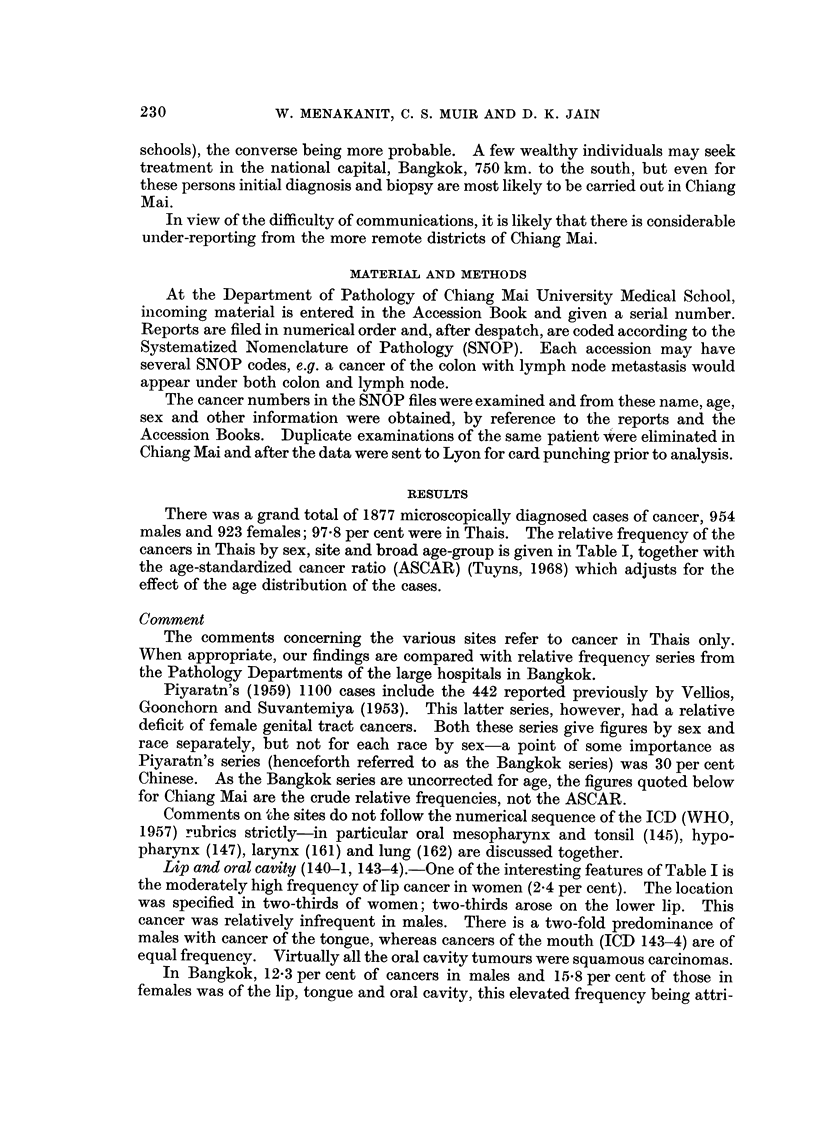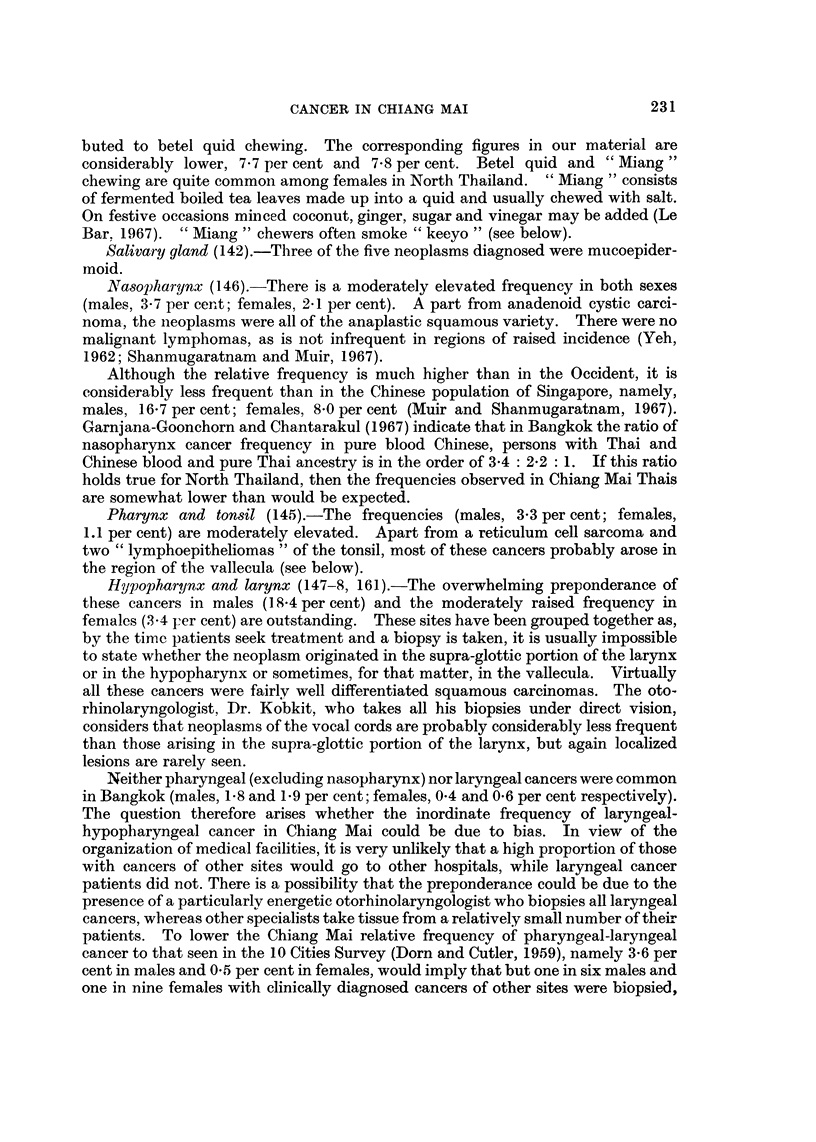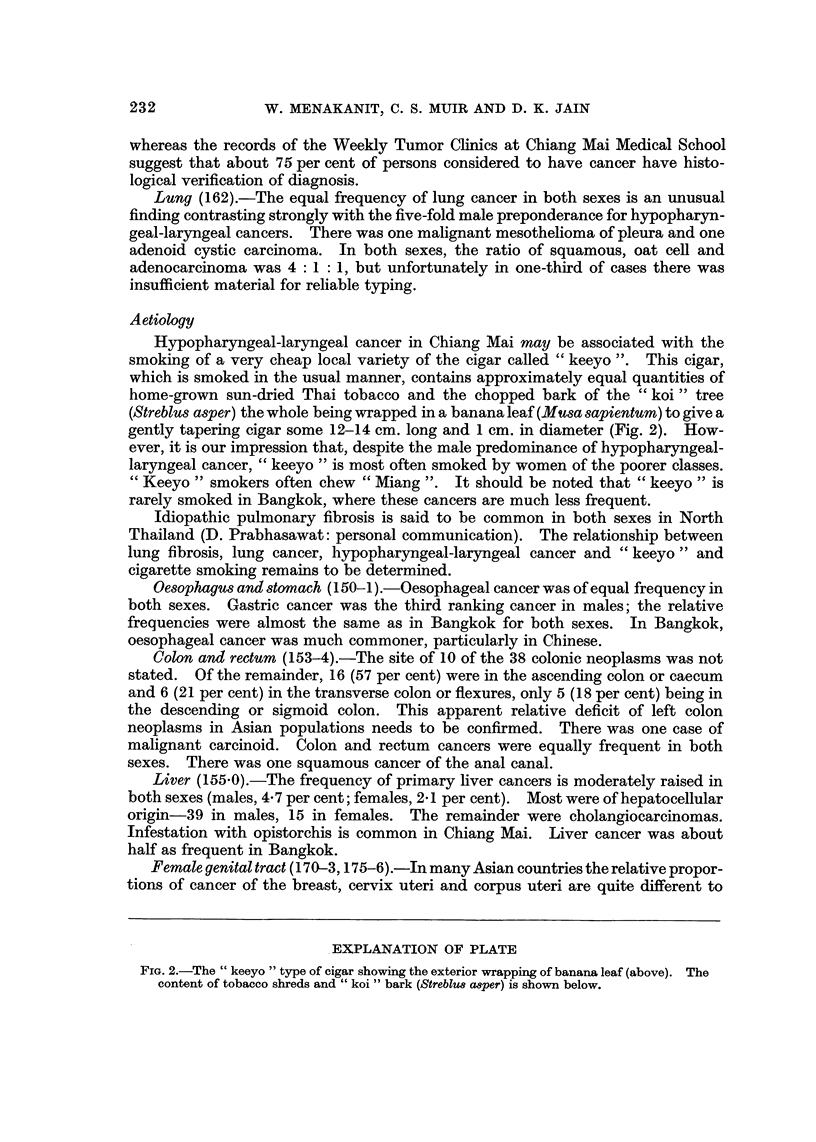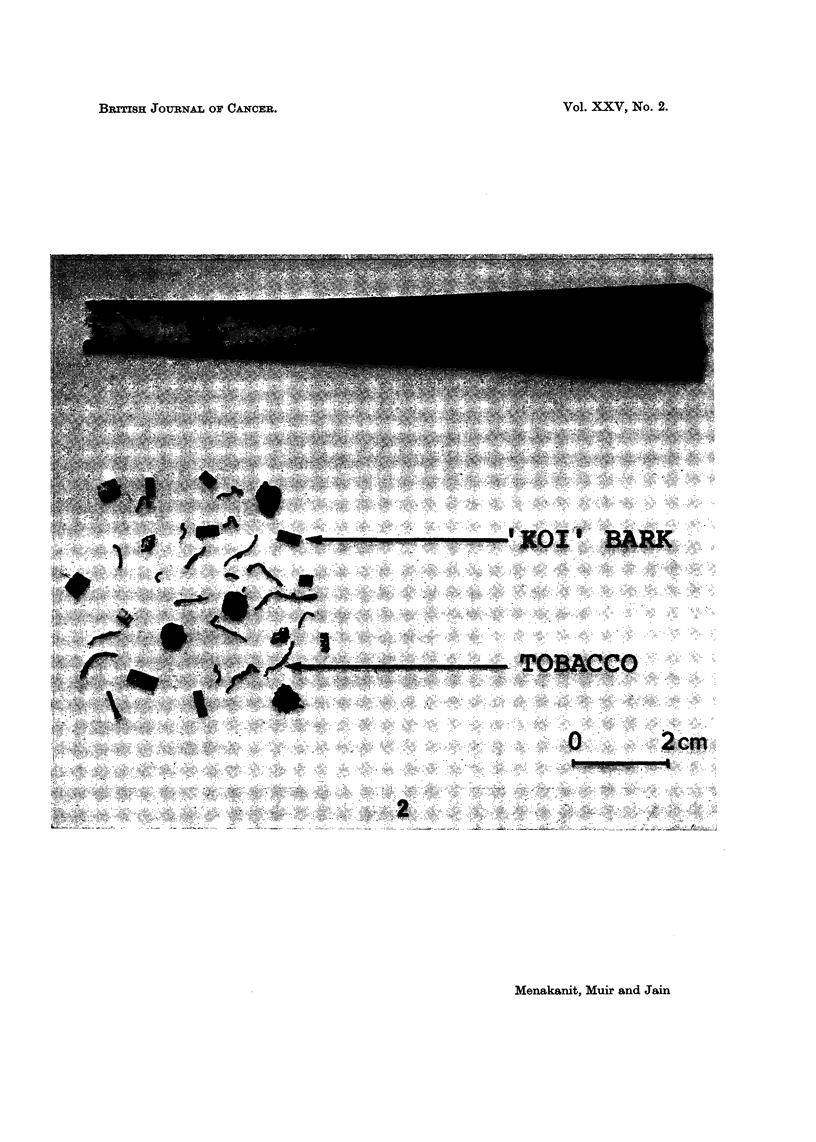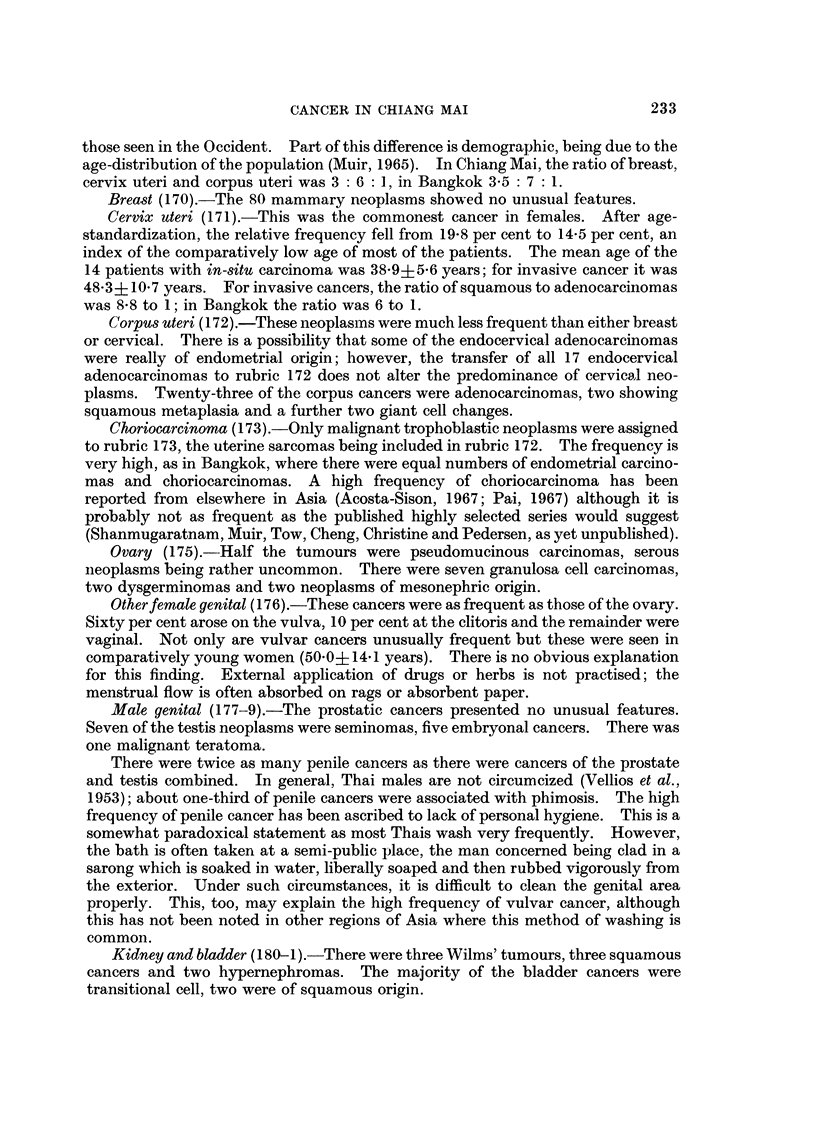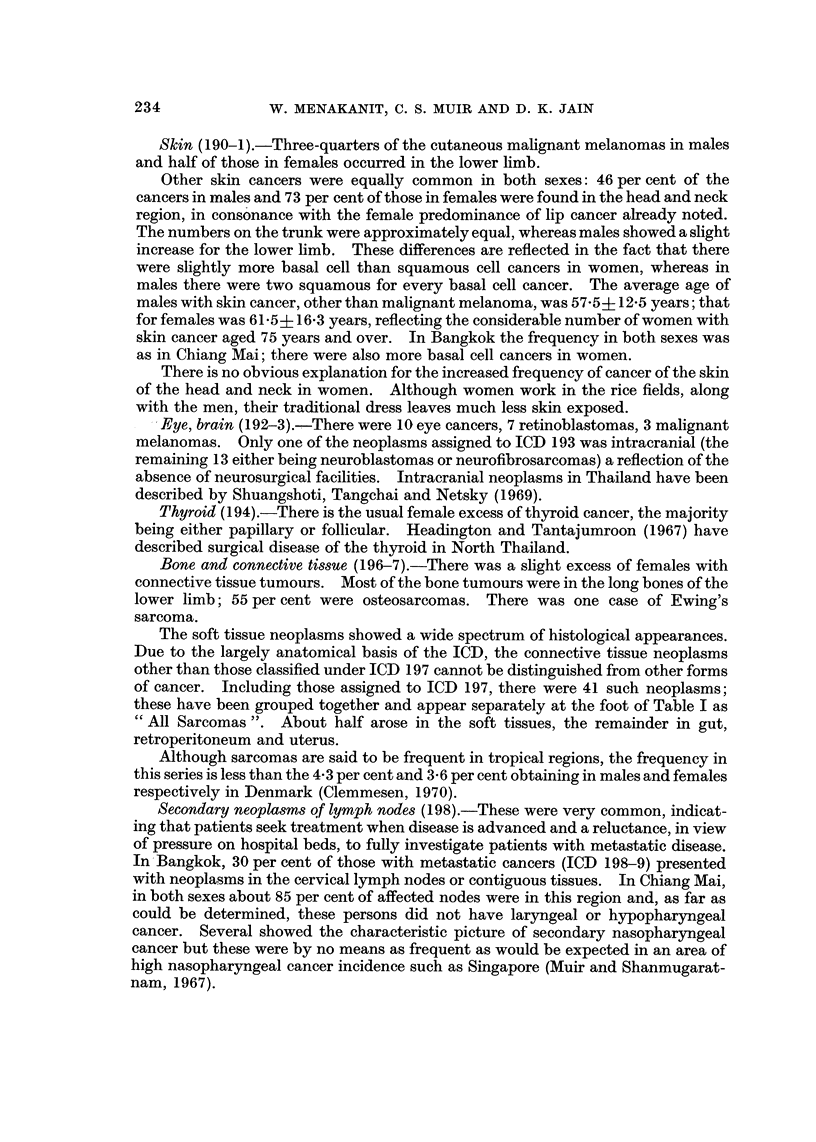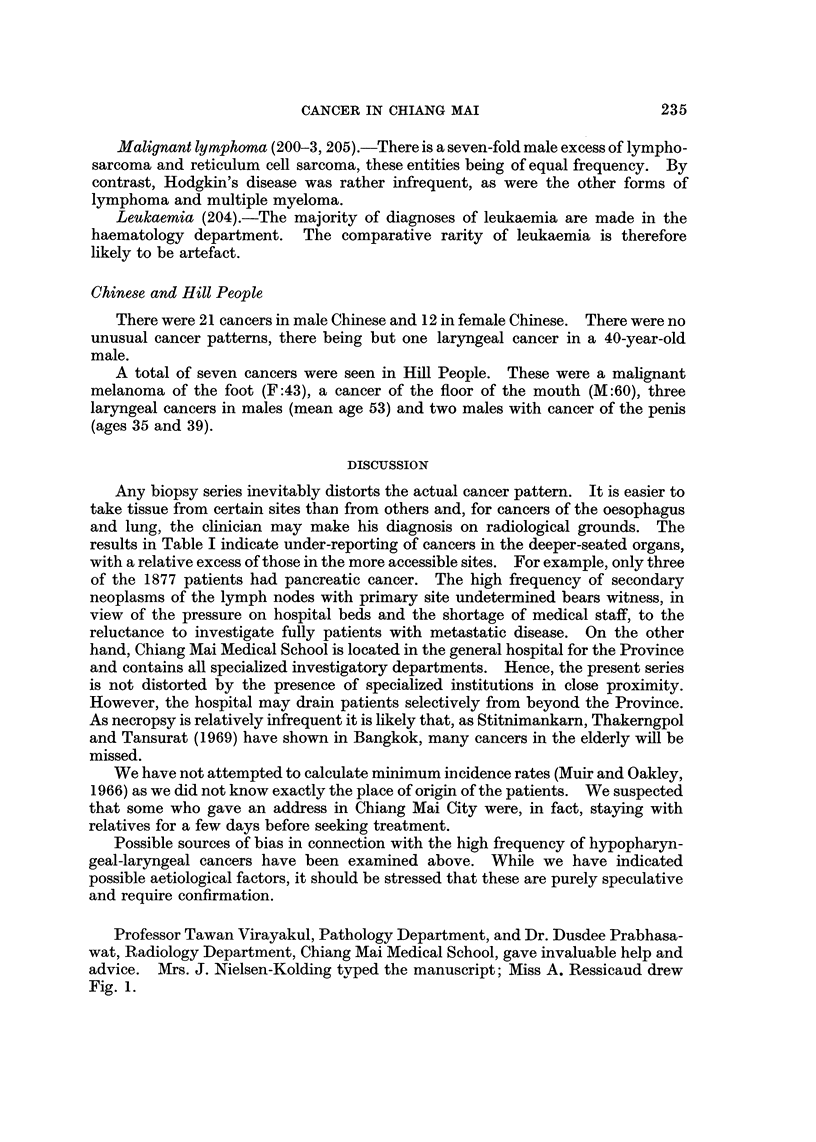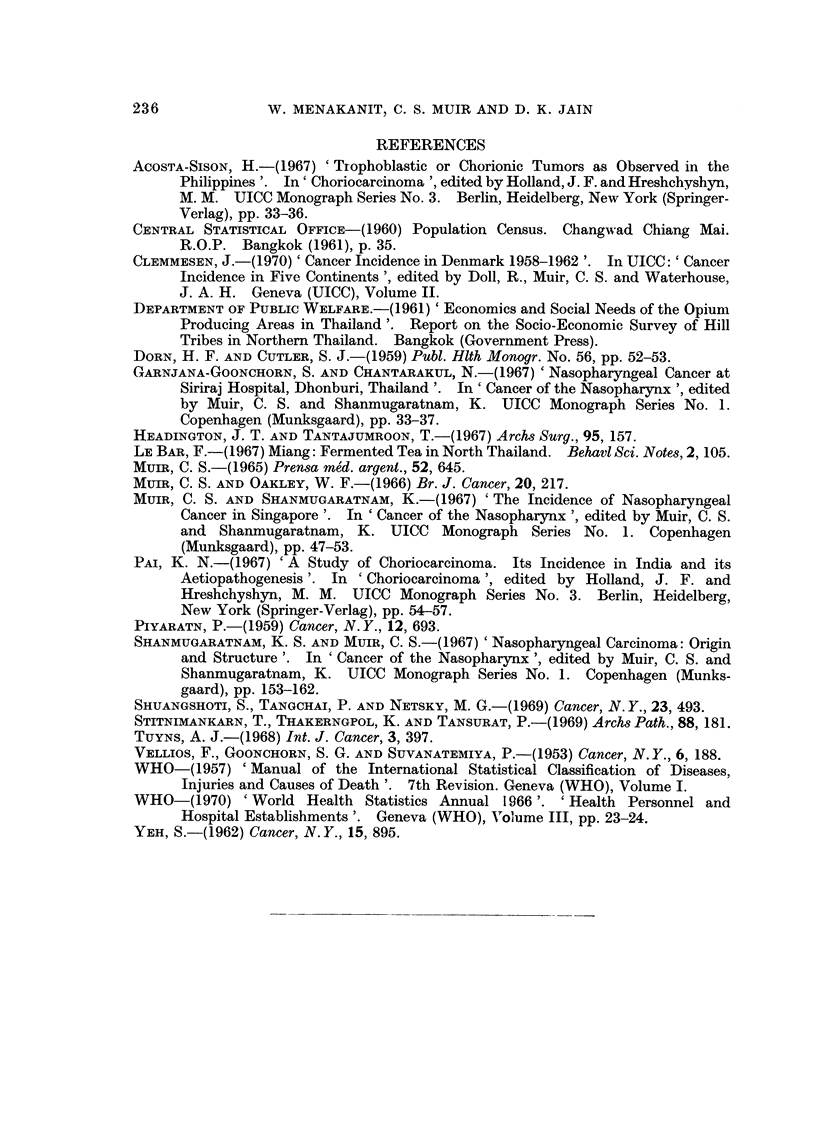# Cancer in Chiang Mai, North Thailand. A Relative Frequency Study

**DOI:** 10.1038/bjc.1971.29

**Published:** 1971-06

**Authors:** W. Menakanit, C. S. Muir, D. K. Jain

## Abstract

**Images:**


BRITISH JOURNAL OF CANCER

VOL. XXV JUNE, 1971 NO. 2

### CANCER IN CHIANG MAI, NORTH THAILAND. A RELATIVE FREQUENCY STUDY

W. MENAKANIT, C. S. MUIR AND D. K. JAIN

*From the Department of Pathology, Chiang Mai Medical School, Chiang Mai, Thailand, and the Epidemiology Unit, International Agency for Research on Cancer, 16 Avenue Maréchal Foch, 69 Lyon 6e, France*

Received for publication October 20, 1970

**SUMMARY.—The relative frequency of histologically diagnosed cancer at the Chiang Mai Medical School in North Thailand in 1964–67 inclusive is examined.**

**Most of the 1877 cancers seen are in Thais (males, 927; females, 908), the remainder arising in Chinese (males, 21; females, 12), Hill People (males, 6; females, 1) and others (females, 2). The cancers in Thais are presented by site, sex and 10-year age-group together with the relative frequency (crude and corrected for age).**

**In Thais, the outstanding finding is the extraordinarily high frequency of cancer of the hypopharyngeal-laryngeal region in both sexes (males, 18·4 per cent; females, 3·4 per cent). This may be associated with the smoking of a local variety of the cigar called " keeyo ". This cigar, smoked in the usual manner, contains approximately equal quantities of home-grown, sun-dried, Thai tobacco and the chopped bark of the " koi " tree (*Streblus asper*). In women, who also smoke " keeyo ", the frequency of hypopharyngeal-laryngeal cancer is unusually high by occidental standards. There is no sex difference in the frequency of bronchial cancer (4 per cent).**

**In males cancer of the penis, in second rank (6·6 per cent) is much more frequent than cancer of the prostate and testis combined. Stomach and skin cancers (ICD 191) are in third place (each 5·7 per cent).**

**In females the most frequent cancers are cervix uteri (19·8 per cent), breast (8·8 per cent) and skin (ICD 191) (6·1 per cent). Cancers of the lip and skin of the head and neck are more frequent in females than in males. Choriocarcinoma is common (1·9 per cent) and there is a large number of vulvar cancers in young women (2·6 per cent).**

**The geography, economy and medical facilities of Chiang Mai Province are described. It is considered that, although there is likely to be considerable under-reporting of internal cancers, the high frequency of hypopharyngeal-laryngeal cancer is not due to selective bias.**

LITTLE is known about cancer in North Thailand. This communication examines the relative frequency of the 1877 cancers diagnosed histologically at the Medical School Chiang Mai in the 4 years 1964–67. In Thais, the commonest cancers in rank order in males were those arising in the laryngeal-hypopharyngeal region, the penis, stomach and skin; in females, cervix uteri, breast and skin. Possible aetiological factors, notably for the laryngeal-hypopharyngeal malignancies, are discussed.

*Chiang Mai Province*

Chiang Mai Province extends over 22,991 km$^2$ in the North of Thailand. The Burmese, Laotian and Chinese borders lie to the north-west and north-east. The 1960 Census population was 798,483 (Central Statistical Office, 1960): the Province is divided into 17 Amphor, the largest, Amphor Muang, having a population of 100,158. The principal city (population 65,600), also called Chiang Mai, is situated in Amphor Muang (Fig. 1).

Chiang Mai is the fourth largest city in Thailand and was formerly the capital of the northern territory. Today, it is the regional capital for the nine North Thai provinces, being the religious, medical, economic, cultural, historical, educational and transportation centre. Situated on the Ping River, it is near the centre of a fertile intermontane basin some 4150 km$^2$ in extent, covered with the so-called Chiang Mai loam. The principal wet season crop is rice; soya beans, peanuts,

**Fig. 1.—Map of Thailand and surrounding countries indicating the position of Chiang Mai.**

tobacco, garlic, onions, pepper, other vegetables and fruit, notably longan, are also grown. Surrounding the basin are mixed deciduous forests which include important teak stands.

Industry is not well-developed, being mainly of the cottage variety—umbrella-making, woodcarving, silversmithing, pottery manufacture and silk weaving.

*The population*

The traditions, costumes and etiquette of the North Thai people are rather different from those of the South.

The population of the Province comprises Thais and Chinese, respectively 99·5 per cent and 0·4 per cent at the 1960 Census. The Thai race is known to have originated in and migrated from the South of China, and Thailand has long been a favourite place of migration among the Chinese. Intermarriage is common. Since many Chinese have assumed Thai names, and would normally describe themselves as Thai (Garnjana-Goonchorn and Chantarakul, 1967) only those who described themselves as Chinese would be so recorded in this communication.

Around Chiang Mai there are also about 25,000 Hill People who, in general, live above 600 m. The principal groups are Shan, Shahleh, Lahu, Lisu, Yao, Lawa, Miao and Karen. The Hill tribes live in small villages or groups and they too have their own distinctive costume and culture. They cultivate rice and poppies, and usually have some livestock; some are semi-nomadic (Department of Public Welfare, 1961). Such individuals are rarely seen in the cities. Medically, comparatively little is known about the Hill People who dwell around Chiang Mai as it is only recently that there have been systematic efforts to bring medical services to this group.

*Statement of age.*—The statements of age are likely to be fairly reliable, with the possible exception of the persons aged 65 years and over. Examination of the stated ages indicates, however, that there was an excess of ages ending in 0 and 5 for both sexes, 126 per cent and 121 per cent of expectation respectively. The age-groups used in Table I would tend to smooth out such distortions.

*Medical facilities*

There are approximately 235 physicians in Chiang Mai Province; 145 in the Medical School and Public Service, 90 in private practice. The ratio of one physician per 3400 population is considerably more favourable than that given by WHO for all Thailand (WHO, 1970).

The University of Chiang Mai Medical School opened in 1960 in the grounds of the provincial hospital. There has been rapid expansion and in the period covered by the survey there were approximately 600 beds. Other hospitals in the city are the McCormick American Presbyterian Mission (300 beds), Chiang Mai Leprosarium (90 beds), Chinda Hospital (30 beds) and two 25-bed private hospitals.

The McCormick Hospital processes its own biopsy specimens and those sent to it from other Presbyterian hospitals in neighbouring provinces, as well as from the Leprosarium and Chinda Hospital. However, a pathologist from the Chiang Mai Medical School interprets this material, which is included in Table I. Radiotherapy is only available at the Medical School.

It is not considered likely that patients would leave Chiang Mai Province to go to hospitals in the neighbouring provincial capitals (which do not have medical

TABLE I.—*Histologically Diagnosed Cancers in Chiang Mai*, 1964–67, *by Age and Sex, with Relative Frequency and ASCAR—Thai Males*

| Site† | ICD 7th† | Age-group 0–4 | 5–24 | 25–34 | 35–44 | 45–54 | 55–64 | 65–74 | 75+ | N.S. | Total | Rel. freq. | ASCAR |
|---|---|---|---|---|---|---|---|---|---|---|---|---|---|
| Lip | 140 | 0 | 0 | 0 | 0 | 1 | 4 | 2 | 0 | 0 | 7 | 0·8 | 0·6 |
| Tongue | 141 | 0 | 0 | 0 | 1 | 8 | 10 | 8 | 2 | 0 | 29 | 3·1 | 3·0 |
| Salivary gland | 142 | 0 | 1 | 1 | 0 | 0 | 2 | 0 | 0 | 0 | 4 | 0·4 | 0·5 |
| Mouth | 143–4 | 0 | 0 | 1 | 1 | 4 | 11 | 12 | 6 | 0 | 35 | 3·8 | 4·4 |
| Nasopharynx | 146 | 0 | 1 | 2 | 9 | 8 | 8 | 5 | 1 | 0 | 34 | 3·7 | 3·3 |
| Pharynx | 145 | 0 | 0 | 0 | 4 | 3 | 11 | 9 | 4 | 0 | 31 | 3·3 | 3·6 |
| Hypopharynx, Larynx | 147–8, 161 | 0 | 1 | 3 | 6 | 22 | 57 | 63 | 19 | 0 | 171 | 18·4 | 19·8 |
| Oesophagus | 150 | 0 | 0 | 0 | 2 | 3 | 7 | 3 | 0 | 0 | 15 | 1·6 | 1·3 |
| Stomach | 151 | 0 | 0 | 2 | 9 | 13 | 21 | 7 | 1 | 0 | 53 | 5·7 | 4·7 |
| Colon | 153 | 0 | 0 | 3 | 4 | 5 | 3 | 6 | 0 | 0 | 21 | 2·3 | 2·0 |
| Rectum | 154 | 0 | 1 | 1 | 0 | 4 | 8 | 6 | 1 | 0 | 21 | 2·3 | 2·3 |
| Liver | 155 | 0 | 0 | 3 | 10 | 15 | 10 | 5 | 1 | 0 | 44 | 4·7 | 4·2 |
| Nose, etc. | 160 | 0 | 0 | 2 | 2 | 2 | 5 | 4 | 0 | 0 | 15 | 1·6 | 1·4 |
| Lung | 162 | 0 | 0 | 2 | 6 | 12 | 8 | 9 | 0 | 0 | 37 | 4·0 | 3·4 |
| Breast | 170 | 0 | 0 | 0 | 1 | 2 | 0 | 0 | 0 | 0 | 3 | 0·3 | 0·2 |
| Prostate | 177 | 0 | 0 | 0 | 0 | 1 | 4 | 8 | 5 | 0 | 18 | 1·9 | 2·7 |
| Testis | 178 | 2 | 2 | 6 | 1 | 2 | 0 | 0 | 0 | 0 | 13 | 1·4 | 1·8 |
| Penis | 179·0 | 0 | 0 | 4 | 14 | 19 | 18 | 5 | 0 | 0 | 60 | 6·6 | 5·2 |
| Kidney, Bladder | 180–1 | 3 | 1 | 0 | 0 | 3 | 2 | 1 | 2 | 0 | 12 | 1·3 | 1·9 |
| Mal. melanoma | 190 | 0 | 0 | 1 | 2 | 3 | 3 | 4 | 3 | 0 | 16 | 1·7 | 2·0 |
| Other skin | 191 | 0 | 1 | 0 | 10 | 10 | 16 | 10 | 6 | 0 | 53 | 5·7 | 5·9 |
| Eye, Brain | 192–3 | 8 | 4 | 0 | 1 | 0 | 0 | 0 | 0 | 0 | 13 | 1·4 | 2·7 |
| Thyroid, etc. | 194–5 | 0 | 1 | 1 | 2 | 2 | 1 | 0 | 0 | 0 | 7 | 0·8 | 0·7 |
| Bone | 196 | 0 | 4 | 3 | 3 | 3 | 0 | 0 | 0 | 0 | 13 | 1·4 | 1·7 |
| Conn. tissue | 197 | 0 | 3 | 0 | 3 | 0 | 1 | 2 | 1 | 0 | 10 | 1·1 | 1·5 |
| Sec. lymph node | 198 | 0 | 1 | 7 | 10 | 21 | 27 | 14 | 2 | 0 | 82 | 8·9 | 7·7 |
| Lymphosarcoma | 200 | 0 | 4 | 4 | 8 | 8 | 4 | 7 | 1 | 0 | 36 | 3·9 | 4·0 |
| Hodgkins, etc. | 201–5 | 0 | 2 | 2 | 2 | 1 | 1 | 1 | 0 | 0 | 9 | 1·0 | 1·1 |
| Others | — | 2 | 2 | 2 | 11 | 17 | 13 | 16 | 2 | 0 | 65 | 7·0 | 6·4 |
| Total | 140–205 | 15 | 29 | 50 | 122 | 192 | 255 | 207 | 57 | 0 | 927 | 100·0 | 100·0 |
| All Sarcomas* | | 1 | 3 | 0 | 3 | 0 | 1 | 3 | 1 | 0 | 12 | 1·3 | 1·9 |

* All these tumours already appear in the body of the table—see text.
† (WHO, 1957).

TABLE I (*Continued*)—*Thai Females*

| Site† | ICD 7th† | Age-group 0–4 | 5–24 | 25–34 | 35–44 | 45–54 | 55–64 | 65–74 | 75+ | N.S. | Total | Rel. freq. | ASCAR |
|---|---|---|---|---|---|---|---|---|---|---|---|---|---|
| Lip | 140 | 0 | 0 | 0 | 1 | 2 | 6 | 9 | 4 | 0 | 22 | 2·4 | 3·8 |
| Tongue | 141 | 0 | 1 | 0 | 0 | 5 | 3 | 4 | 1 | 0 | 14 | 1·5 | 2·0 |
| Salivary gland | 142 | 0 | 0 | 0 | 0 | 0 | 0 | 0 | 0 | 1 | 1 | 0·1 | 0·0 |
| Mouth | 143–4 | 0 | 0 | 1 | 1 | 5 | 14 | 10 | 4 | 0 | 35 | 3·9 | 5·1 |
| Nasopharynx | 146 | 0 | 2 | 4 | 4 | 3 | 3 | 2 | 1 | 0 | 19 | 2·1 | 2·1 |
| Pharynx, etc. | 145 | 0 | 0 | 0 | 1 | 2 | 4 | 3 | 0 | 0 | 10 | 1·1 | 1·2 |
| Hypopharynx, larynx | 147–8, 161 | 0 | 0 | 0 | 2 | 10 | 10 | 7 | 2 | 0 | 31 | 3·4 | 3·8 |
| Oesophagus | 150 | 0 | 0 | 0 | 2 | 2 | 9 | 2 | 0 | 0 | 15 | 1·7 | 1·5 |
| Stomach | 151 | 0 | 1 | 0 | 6 | 8 | 8 | 1 | 1 | 0 | 25 | 2·8 | 2·4 |
| Colon | 153 | 0 | 0 | 3 | 4 | 5 | 3 | 2 | 0 | 0 | 17 | 1·9 | 1·4 |
| Rectum | 154 | 0 | 1 | 2 | 3 | 4 | 3 | 3 | 1 | 0 | 17 | 1·9 | 2·0 |
| Liver | 155 | 0 | 0 | 1 | 4 | 3 | 6 | 4 | 1 | 0 | 19 | 2·1 | 2·0 |
| Nose, etc. | 160 | 0 | 0 | 2 | 0 | 0 | 0 | 2 | 0 | 1 | 5 | 0·6 | 0·5 |
| Lung | 162 | 0 | 0 | 2 | 7 | 19 | 5 | 4 | 1 | 0 | 38 | 4·2 | 3·4 |
| Breast | 170 | 0 | 0 | 5 | 25 | 21 | 16 | 9 | 3 | 1 | 80 | 8·8 | 7·4 |
| Cervix | 171 | 0 | 1 | 14 | 64 | 51 | 34 | 15 | 1 | 0 | 180 | 19·8 | 14·5 |
| Corpus | 172 | 0 | 2 | 2 | 7 | 7 | 7 | 4 | 0 | 0 | 29 | 3·2 | 2·9 |
| Choriocarcinoma | 173 | 0 | 1 | 4 | 10 | 2 | 0 | 0 | 0 | 0 | 17 | 1·9 | 1·1 |
| Ovary | 175 | 1 | 4 | 8 | 9 | 12 | 4 | 0 | 2 | 0 | 40 | 4·4 | 4·1 |
| Fem. genit. | 176 | 1 | 0 | 4 | 13 | 8 | 5 | 8 | 1 | 0 | 40 | 4·4 | 4·1 |
| Kidney, Bladder | 180–1 | 0 | 0 | 0 | 3 | 3 | 6 | 3 | 1 | 0 | 16 | 1·8 | 1·9 |
| Mal. melanoma | 190 | 0 | 1 | 0 | 0 | 3 | 2 | 2 | 0 | 1 | 9 | 1·0 | 1·0 |
| Other skin | 191 | 0 | 1 | 3 | 6 | 7 | 12 | 12 | 14 | 0 | 55 | 6·1 | 9·5 |
| Eye, Brain | 192–3 | 4 | 1 | 1 | 1 | 3 | 1 | 0 | 0 | 0 | 11 | 1·2 | 2·0 |
| Thyroid, etc. | 194–5 | 0 | 1 | 6 | 0 | 3 | 2 | 6 | 1 | 0 | 19 | 2·1 | 2·5 |
| Bone | 196 | 0 | 4 | 1 | 1 | 0 | 0 | 1 | 0 | 0 | 7 | 0·8 | 1·5 |
| Conn, tissue | 197 | 3 | 3 | 3 | 5 | 1 | 3 | 0 | 0 | 0 | 18 | 2·0 | 2·6 |
| Sec. lymph node | 198 | 0 | 1 | 3 | 12 | 11 | 13 | 12 | 0 | 0 | 52 | 5·7 | 5·3 |
| Lymphosarcoma | 200 | 0 | 1 | 1 | 1 | 0 | 0 | 2 | 0 | 0 | 5 | 0·6 | 0·7 |
| Hodgkins, etc. | 201–5 | 0 | 1 | 0 | 1 | 2 | 1 | 0 | 0 | 0 | 5 | 0·6 | 0·5 |
| Others | — | 1 | 1 | 2 | 9 | 14 | 18 | 7 | 5 | 0 | 57 | 6·3 | 7·3 |
| Total | 140–205 | 10 | 28 | 72 | 202 | 216 | 198 | 134 | 44 | 4 | 908 | 100·0 | 100·0 |
| All Sarcomas* | | 4 | 3 | 3 | 8 | 4 | 5 | 1 | 1 | 0 | 29 | 3·2 | 3·9 |

* All these tumours already appear in the body of the table—see text.
† (WHO, 1957).

schools), the converse being more probable. A few wealthy individuals may seek treatment in the national capital, Bangkok, 750 km. to the south, but even for these persons initial diagnosis and biopsy are most likely to be carried out in Chiang Mai.

In view of the difficulty of communications, it is likely that there is considerable under-reporting from the more remote districts of Chiang Mai.

### MATERIAL AND METHODS

At the Department of Pathology of Chiang Mai University Medical School, incoming material is entered in the Accession Book and given a serial number. Reports are filed in numerical order and, after despatch, are coded according to the Systematized Nomenclature of Pathology (SNOP). Each accession may have several SNOP codes, *e.g.* a cancer of the colon with lymph node metastasis would appear under both colon and lymph node.

The cancer numbers in the SNOP files were examined and from these name, age, sex and other information were obtained, by reference to the reports and the Accession Books. Duplicate examinations of the same patient were eliminated in Chiang Mai and after the data were sent to Lyon for card punching prior to analysis.

### RESULTS

There was a grand total of 1877 microscopically diagnosed cases of cancer, 954 males and 923 females; 97·8 per cent were in Thais. The relative frequency of the cancers in Thais by sex, site and broad age-group is given in Table I, together with the age-standardized cancer ratio (ASCAR) (Tuyns, 1968) which adjusts for the effect of the age distribution of the cases.

### *Comment*

The comments concerning the various sites refer to cancer in Thais only. When appropriate, our findings are compared with relative frequency series from the Pathology Departments of the large hospitals in Bangkok.

Piyaratn's (1959) 1100 cases include the 442 reported previously by Vellios, Goonchorn and Suvantemiya (1953). This latter series, however, had a relative deficit of female genital tract cancers. Both these series give figures by sex and race separately, but not for each race by sex—a point of some importance as Piyaratn's series (henceforth referred to as the Bangkok series) was 30 per cent Chinese. As the Bangkok series are uncorrected for age, the figures quoted below for Chiang Mai are the crude relative frequencies, not the ASCAR.

Comments on the sites do not follow the numerical sequence of the ICD (WHO, 1957) rubrics strictly—in particular oral mesopharynx and tonsil (145), hypopharynx (147), larynx (161) and lung (162) are discussed together.

*Lip and oral cavity* (140–1, 143–4).—One of the interesting features of Table I is the moderately high frequency of lip cancer in women (2·4 per cent). The location was specified in two-thirds of women; two-thirds arose on the lower lip. This cancer was relatively infrequent in males. There is a two-fold predominance of males with cancer of the tongue, whereas cancers of the mouth (ICD 143–4) are of equal frequency. Virtually all the oral cavity tumours were squamous carcinomas.

In Bangkok, 12·3 per cent of cancers in males and 15·8 per cent of those in females was of the lip, tongue and oral cavity, this elevated frequency being attributed to betel quid chewing. The corresponding figures in our material are considerably lower, 7·7 per cent and 7·8 per cent. Betel quid and " Miang " chewing are quite common among females in North Thailand. " Miang " consists of fermented boiled tea leaves made up into a quid and usually chewed with salt. On festive occasions minced coconut, ginger, sugar and vinegar may be added (Le Bar, 1967). " Miang " chewers often smoke " keeyo " (see below).

*Salivary gland* (142).—Three of the five neoplasms diagnosed were mucoepidermoid.

*Nasopharynx* (146).—There is a moderately elevated frequency in both sexes (males, 3·7 per cent; females, 2·1 per cent). A part from anadenoid cystic carcinoma, the neoplasms were all of the anaplastic squamous variety. There were no malignant lymphomas, as is not infrequent in regions of raised incidence (Yeh, 1962; Shanmugaratnam and Muir, 1967).

Although the relative frequency is much higher than in the Occident, it is considerably less frequent than in the Chinese population of Singapore, namely, males, 16·7 per cent; females, 8·0 per cent (Muir and Shanmugaratnam, 1967). Garnjana-Goonchorn and Chantarakul (1967) indicate that in Bangkok the ratio of nasopharynx cancer frequency in pure blood Chinese, persons with Thai and Chinese blood and pure Thai ancestry is in the order of 3·4 : 2·2 : 1. If this ratio holds true for North Thailand, then the frequencies observed in Chiang Mai Thais are somewhat lower than would be expected.

*Pharynx and tonsil* (145).—The frequencies (males, 3·3 per cent; females, 1.1 per cent) are moderately elevated. Apart from a reticulum cell sarcoma and two " lymphoepitheliomas " of the tonsil, most of these cancers probably arose in the region of the vallecula (see below).

*Hypopharynx and larynx* (147–8, 161).—The overwhelming preponderance of these cancers in males (18·4 per cent) and the moderately raised frequency in females (3·4 per cent) are outstanding. These sites have been grouped together as, by the time patients seek treatment and a biopsy is taken, it is usually impossible to state whether the neoplasm originated in the supra-glottic portion of the larynx or in the hypopharynx or sometimes, for that matter, in the vallecula. Virtually all these cancers were fairly well differentiated squamous carcinomas. The otorhinolaryngologist, Dr. Kobkit, who takes all his biopsies under direct vision, considers that neoplasms of the vocal cords are probably considerably less frequent than those arising in the supra-glottic portion of the larynx, but again localized lesions are rarely seen.

Neither pharyngeal (excluding nasopharynx) nor laryngeal cancers were common in Bangkok (males, 1·8 and 1·9 per cent; females, 0·4 and 0·6 per cent respectively). The question therefore arises whether the inordinate frequency of laryngeal-hypopharyngeal cancer in Chiang Mai could be due to bias. In view of the organization of medical facilities, it is very unlikely that a high proportion of those with cancers of other sites would go to other hospitals, while laryngeal cancer patients did not. There is a possibility that the preponderance could be due to the presence of a particularly energetic otorhinolaryngologist who biopsies all laryngeal cancers, whereas other specialists take tissue from a relatively small number of their patients. To lower the Chiang Mai relative frequency of pharyngeal-laryngeal cancer to that seen in the 10 Cities Survey (Dorn and Cutler, 1959), namely 3·6 per cent in males and 0·5 per cent in females, would imply that but one in six males and one in nine females with clinically diagnosed cancers of other sites were biopsied, whereas the records of the Weekly Tumor Clinics at Chiang Mai Medical School suggest that about 75 per cent of persons considered to have cancer have histological verification of diagnosis.

*Lung* (162).—The equal frequency of lung cancer in both sexes is an unusual finding contrasting strongly with the five-fold male preponderance for hypopharyngeal-laryngeal cancers. There was one malignant mesothelioma of pleura and one adenoid cystic carcinoma. In both sexes, the ratio of squamous, oat cell and adenocarcinoma was 4 : 1 : 1, but unfortunately in one-third of cases there was insufficient material for reliable typing.

*Aetiology*

Hypopharyngeal-laryngeal cancer in Chiang Mai *may* be associated with the smoking of a very cheap local variety of the cigar called " keeyo ". This cigar, which is smoked in the usual manner, contains approximately equal quantities of home-grown sun-dried Thai tobacco and the chopped bark of the " koi " tree (*Streblus asper*) the whole being wrapped in a banana leaf (*Musa sapientum*) to give a gently tapering cigar some 12–14 cm. long and 1 cm. in diameter (Fig. 2). However, it is our impression that, despite the male predominance of hypopharyngeal-laryngeal cancer, " keeyo " is most often smoked by women of the poorer classes. " Keeyo " smokers often chew " Miang ". It should be noted that " keeyo " is rarely smoked in Bangkok, where these cancers are much less frequent.

Idiopathic pulmonary fibrosis is said to be common in both sexes in North Thailand (D. Prabhasawat: personal communication). The relationship between lung fibrosis, lung cancer, hypopharyngeal-laryngeal cancer and " keeyo " and cigarette smoking remains to be determined.

*Oesophagus and stomach* (150–1).—Oesophageal cancer was of equal frequency in both sexes. Gastric cancer was the third ranking cancer in males; the relative frequencies were almost the same as in Bangkok for both sexes. In Bangkok, oesophageal cancer was much commoner, particularly in Chinese.

*Colon and rectum* (153–4).—The site of 10 of the 38 colonic neoplasms was not stated. Of the remainder, 16 (57 per cent) were in the ascending colon or caecum and 6 (21 per cent) in the transverse colon or flexures, only 5 (18 per cent) being in the descending or sigmoid colon. This apparent relative deficit of left colon neoplasms in Asian populations needs to be confirmed. There was one case of malignant carcinoid. Colon and rectum cancers were equally frequent in both sexes. There was one squamous cancer of the anal canal.

*Liver* (155·0).—The frequency of primary liver cancers is moderately raised in both sexes (males, 4·7 per cent; females, 2·1 per cent). Most were of hepatocellular origin—39 in males, 15 in females. The remainder were cholangiocarcinomas. Infestation with opistorchis is common in Chiang Mai. Liver cancer was about half as frequent in Bangkok.

*Female genital tract* (170–3, 175–6).—In many Asian countries the relative proportions of cancer of the breast, cervix uteri and corpus uteri are quite different to

---

EXPLANATION OF PLATE

FIG. 2.—The " keeyo " type of cigar showing the exterior wrapping of banana leaf (above). The content of tobacco shreds and " koi " bark (*Streblus asper*) is shown below.

Menakanit, Muir and Jain those seen in the Occident. Part of this difference is demographic, being due to the age-distribution of the population (Muir, 1965). In Chiang Mai, the ratio of breast, cervix uteri and corpus uteri was 3 : 6 : 1, in Bangkok 3·5 : 7 : 1.

*Breast* (170).—The 80 mammary neoplasms showed no unusual features.

*Cervix uteri* (171).—This was the commonest cancer in females. After age-standardization, the relative frequency fell from 19·8 per cent to 14·5 per cent, an index of the comparatively low age of most of the patients. The mean age of the 14 patients with *in-situ* carcinoma was 38·9$\pm$5·6 years; for invasive cancer it was 48·3$\pm$10·7 years. For invasive cancers, the ratio of squamous to adenocarcinomas was 8·8 to 1; in Bangkok the ratio was 6 to 1.

*Corpus uteri* (172).—These neoplasms were much less frequent than either breast or cervical. There is a possibility that some of the endocervical adenocarcinomas were really of endometrial origin; however, the transfer of all 17 endocervical adenocarcinomas to rubric 172 does not alter the predominance of cervical neoplasms. Twenty-three of the corpus cancers were adenocarcinomas, two showing squamous metaplasia and a further two giant cell changes.

*Choriocarcinoma* (173).—Only malignant trophoblastic neoplasms were assigned to rubric 173, the uterine sarcomas being included in rubric 172. The frequency is very high, as in Bangkok, where there were equal numbers of endometrial carcinomas and choriocarcinomas. A high frequency of choriocarcinoma has been reported from elsewhere in Asia (Acosta-Sison, 1967; Pai, 1967) although it is probably not as frequent as the published highly selected series would suggest (Shanmugaratnam, Muir, Tow, Cheng, Christine and Pedersen, as yet unpublished).

*Ovary* (175).—Half the tumours were pseudomucinous carcinomas, serous neoplasms being rather uncommon. There were seven granulosa cell carcinomas, two dysgerminomas and two neoplasms of mesonephric origin.

*Other female genital* (176).—These cancers were as frequent as those of the ovary. Sixty per cent arose on the vulva, 10 per cent at the clitoris and the remainder were vaginal. Not only are vulvar cancers unusually frequent but these were seen in comparatively young women (50·0$\pm$14·1 years). There is no obvious explanation for this finding. External application of drugs or herbs is not practised; the menstrual flow is often absorbed on rags or absorbent paper.

*Male genital* (177–9).—The prostatic cancers presented no unusual features. Seven of the testis neoplasms were seminomas, five embryonal cancers. There was one malignant teratoma.

There were twice as many penile cancers as there were cancers of the prostate and testis combined. In general, Thai males are not circumcized (Vellios *et al.*, 1953); about one-third of penile cancers were associated with phimosis. The high frequency of penile cancer has been ascribed to lack of personal hygiene. This is a somewhat paradoxical statement as most Thais wash very frequently. However, the bath is often taken at a semi-public place, the man concerned being clad in a sarong which is soaked in water, liberally soaped and then rubbed vigorously from the exterior. Under such circumstances, it is difficult to clean the genital area properly. This, too, may explain the high frequency of vulvar cancer, although this has not been noted in other regions of Asia where this method of washing is common.

*Kidney and bladder* (180–1).—There were three Wilms' tumours, three squamous cancers and two hypernephromas. The majority of the bladder cancers were transitional cell, two were of squamous origin.

*Skin* (190–1).—Three-quarters of the cutaneous malignant melanomas in males and half of those in females occurred in the lower limb.

Other skin cancers were equally common in both sexes: 46 per cent of the cancers in males and 73 per cent of those in females were found in the head and neck region, in consonance with the female predominance of lip cancer already noted. The numbers on the trunk were approximately equal, whereas males showed a slight increase for the lower limb. These differences are reflected in the fact that there were slightly more basal cell than squamous cell cancers in women, whereas in males there were two squamous for every basal cell cancer. The average age of males with skin cancer, other than malignant melanoma, was $57 \cdot 5 \pm 12 \cdot 5$ years; that for females was $61 \cdot 5 \pm 16 \cdot 3$ years, reflecting the considerable number of women with skin cancer aged 75 years and over. In Bangkok the frequency in both sexes was as in Chiang Mai; there were also more basal cell cancers in women.

There is no obvious explanation for the increased frequency of cancer of the skin of the head and neck in women. Although women work in the rice fields, along with the men, their traditional dress leaves much less skin exposed.

*Eye, brain* (192–3).—There were 10 eye cancers, 7 retinoblastomas, 3 malignant melanomas. Only one of the neoplasms assigned to ICD 193 was intracranial (the remaining 13 either being neuroblastomas or neurofibrosarcomas) a reflection of the absence of neurosurgical facilities. Intracranial neoplasms in Thailand have been described by Shuangshoti, Tangchai and Netsky (1969).

*Thyroid* (194).—There is the usual female excess of thyroid cancer, the majority being either papillary or follicular. Headington and Tantajumroon (1967) have described surgical disease of the thyroid in North Thailand.

*Bone and connective tissue* (196–7).—There was a slight excess of females with connective tissue tumours. Most of the bone tumours were in the long bones of the lower limb; 55 per cent were osteosarcomas. There was one case of Ewing's sarcoma.

The soft tissue neoplasms showed a wide spectrum of histological appearances. Due to the largely anatomical basis of the ICD, the connective tissue neoplasms other than those classified under ICD 197 cannot be distinguished from other forms of cancer. Including those assigned to ICD 197, there were 41 such neoplasms; these have been grouped together and appear separately at the foot of Table I as " All Sarcomas ". About half arose in the soft tissues, the remainder in gut, retroperitoneum and uterus.

Although sarcomas are said to be frequent in tropical regions, the frequency in this series is less than the 4·3 per cent and 3·6 per cent obtaining in males and females respectively in Denmark (Clemmesen, 1970).

*Secondary neoplasms of lymph nodes* (198).—These were very common, indicating that patients seek treatment when disease is advanced and a reluctance, in view of pressure on hospital beds, to fully investigate patients with metastatic disease. In Bangkok, 30 per cent of those with metastatic cancers (ICD 198–9) presented with neoplasms in the cervical lymph nodes or contiguous tissues. In Chiang Mai, in both sexes about 85 per cent of affected nodes were in this region and, as far as could be determined, these persons did not have laryngeal or hypopharyngeal cancer. Several showed the characteristic picture of secondary nasopharyngeal cancer but these were by no means as frequent as would be expected in an area of high nasopharyngeal cancer incidence such as Singapore (Muir and Shanmugaratnam, 1967).

*Malignant lymphoma* (200–3, 205).—There is a seven-fold male excess of lymphosarcoma and reticulum cell sarcoma, these entities being of equal frequency. By contrast, Hodgkin's disease was rather infrequent, as were the other forms of lymphoma and multiple myeloma.

*Leukaemia* (204).—The majority of diagnoses of leukaemia are made in the haematology department. The comparative rarity of leukaemia is therefore likely to be artefact.

### *Chinese and Hill People*

There were 21 cancers in male Chinese and 12 in female Chinese. There were no unusual cancer patterns, there being but one laryngeal cancer in a 40-year-old male.

A total of seven cancers were seen in Hill People. These were a malignant melanoma of the foot (F:43), a cancer of the floor of the mouth (M:60), three laryngeal cancers in males (mean age 53) and two males with cancer of the penis (ages 35 and 39).

### DISCUSSION

Any biopsy series inevitably distorts the actual cancer pattern. It is easier to take tissue from certain sites than from others and, for cancers of the oesophagus and lung, the clinician may make his diagnosis on radiological grounds. The results in Table I indicate under-reporting of cancers in the deeper-seated organs, with a relative excess of those in the more accessible sites. For example, only three of the 1877 patients had pancreatic cancer. The high frequency of secondary neoplasms of the lymph nodes with primary site undetermined bears witness, in view of the pressure on hospital beds and the shortage of medical staff, to the reluctance to investigate fully patients with metastatic disease. On the other hand, Chiang Mai Medical School is located in the general hospital for the Province and contains all specialized investigatory departments. Hence, the present series is not distorted by the presence of specialized institutions in close proximity. However, the hospital may drain patients selectively from beyond the Province. As necropsy is relatively infrequent it is likely that, as Stitnimankarn, Thakerngpol and Tansurat (1969) have shown in Bangkok, many cancers in the elderly will be missed.

We have not attempted to calculate minimum incidence rates (Muir and Oakley, 1966) as we did not know exactly the place of origin of the patients. We suspected that some who gave an address in Chiang Mai City were, in fact, staying with relatives for a few days before seeking treatment.

Possible sources of bias in connection with the high frequency of hypopharyngeal-laryngeal cancers have been examined above. While we have indicated possible aetiological factors, it should be stressed that these are purely speculative and require confirmation.

Professor Tawan Virayakul, Pathology Department, and Dr. Dusdee Prabhasawat, Radiology Department, Chiang Mai Medical School, gave invaluable help and advice. Mrs. J. Nielsen-Kolding typed the manuscript; Miss A. Ressicaud drew Fig. 1.

### REFERENCES

ACOSTA-SISON, H.—(1967) ' Tiophoblastic or Chorionic Tumors as Observed in the Philippines '. In ' Choriocarcinoma ', edited by Holland, J. F. and Hreshchyshyn, M. M. UICC Monograph Series No. 3. Berlin, Heidelberg, New York (Springer-Verlag), pp. 33–36.

CENTRAL STATISTICAL OFFICE—(1960) Population Census. Changwad Chiang Mai. R.O.P. Bangkok (1961), p. 35.

CLEMMESEN, J.—(1970) ' Cancer Incidence in Denmark 1958–1962 '. In UICC: ' Cancer Incidence in Five Continents ', edited by Doll, R., Muir, C. S. and Waterhouse, J. A. H. Geneva (UICC), Volume II.

DEPARTMENT OF PUBLIC WELFARE.—(1961) ' Economics and Social Needs of the Opium Producing Areas in Thailand '. Report on the Socio-Economic Survey of Hill Tribes in Northern Thailand. Bangkok (Government Press).

DORN, H. F. AND CUTLER, S. J.—(1959) *Publ. Hlth Monogr.* No. 56, pp. 52–53.

GARNJANA-GOONCHORN, S. AND CHANTARAKUL, N.—(1967) ' Nasopharyngeal Cancer at Siriraj Hospital, Dhonburi, Thailand '. In ' Cancer of the Nasopharynx ', edited by Muir, C. S. and Shanmugaratnam, K. UICC Monograph Series No. 1. Copenhagen (Munksgaard), pp. 33–37.

HEADINGTON, J. T. AND TANTAJUMROON, T.—(1967) *Archs Surg.*, **95**, 157.

LE BAR, F.—(1967) Miang: Fermented Tea in North Thailand. *Behavl Sci. Notes*, **2**, 105.

MUIR, C. S.—(1965) *Prensa méd. argent.*, **52**, 645.

MUIR, C. S. AND OAKLEY, W. F.—(1966) *Br. J. Cancer*, **20**, 217.

MUIR, C. S. AND SHANMUGARATNAM, K.—(1967) ' The Incidence of Nasopharyngeal Cancer in Singapore '. In ' Cancer of the Nasopharynx ', edited by Muir, C. S. and Shanmugaratnam, K. UICC Monograph Series No. 1. Copenhagen (Munksgaard), pp. 47–53.

PAI, K. N.—(1967) ' A Study of Choriocarcinoma. Its Incidence in India and its Aetiopathogenesis '. In ' Choriocarcinoma ', edited by Holland, J. F. and Hreshchyshyn, M. M. UICC Monograph Series No. 3. Berlin, Heidelberg, New York (Springer-Verlag), pp. 54–57.

PIYARATN, P.—(1959) *Cancer, N.Y.*, **12**, 693.

SHANMUGARATNAM, K. S. AND MUIR, C. S.—(1967) ' Nasopharyngeal Carcinoma: Origin and Structure '. In ' Cancer of the Nasopharynx ', edited by Muir, C. S. and Shanmugaratnam, K. UICC Monograph Series No. 1. Copenhagen (Munksgaard), pp. 153–162.

SHUANGSHOTI, S., TANGCHAI, P. AND NETSKY, M. G.—(1969) *Cancer, N.Y.*, **23**, 493.

STITNIMANKARN, T., THAKERNGPOL, K. AND TANSURAT, P.—(1969) *Archs Path.*, **88**, 181.

TUYNS, A. J.—(1968) *Int. J. Cancer*, **3**, 397.

VELLIOS, F., GOONCHORN, S. G. AND SUVANATEMIYA, P.—(1953) *Cancer, N.Y.*, **6**, 188.

WHO—(1957) ' Manual of the International Statistical Classification of Diseases, Injuries and Causes of Death '. 7th Revision. Geneva (WHO), Volume I.

WHO—(1970) ' World Health Statistics Annual 1966 '. ' Health Personnel and Hospital Establishments '. Geneva (WHO), Volume III, pp. 23–24.

YEH, S.—(1962) *Cancer, N.Y.*, **15**, 895.